# Aboulomania, a Mental Disorder Characterized by Pathological Indecisiveness

**DOI:** 10.7759/cureus.41592

**Published:** 2023-07-09

**Authors:** Karlyle Bistas, Jean Paul Tabet

**Affiliations:** 1 Behavioral Health, Wayne State University Detroit Medical Center, Detroit, USA; 2 School of Medicine, Medical University of the Americas, Charlestown, KNA

**Keywords:** depression, anxiety, post-traumatic stress disorder, neuroticism, aboulomania

## Abstract

The mental disorder known as aboulomania, characterized by pathological indecisiveness, is not listed in the Diagnostic and Statistical Manual of Mental Disorders, Fifth Edition, Text Revision (DSM-5-TR), widely used by mental health professionals to diagnose mental illnesses. However, it is frequently observed alongside other mental disorders. Aboulomania is linked to neurotic thinking or "neurosis," which pertains to a mental disorder arising from previous anxiety. This case presentation is on a 40-year-old Caucasian male, with a past psychiatric history of post-traumatic stress disorder (PTSD) and moderate cannabis use disorder, with no known medical history, who was involuntarily admitted to the psychiatric ward. Prolonged hospitalization of over two weeks was attributed to his severe and persistent indecisiveness, which hindered progress in discharge planning. In order to tackle this problem, the patient received encouragement from his treatment team to take small, concrete actions to deal with his indecisiveness. This case report emphasizes the significance of aboulomania in causing long-lasting indecisiveness and provides valuable insights on how to overcome this condition.

## Introduction

Post-traumatic stress disorder (PTSD) is a prevalent mental illness affecting a significant portion of the adult population in the United States and Canada [1-3]. Lifetime prevalence rates range from 6.1% to 9.2%, while one-year prevalence rates fall between 3.5% and 4.7% [1-3]. A recent study conducted in the United States with 5,692 participants revealed that 82.7% of them had experienced severe and traumatic events [4].

PTSD encompasses a wide range of symptoms that may occasionally overlap with diagnostic criteria for other mental health disorders. However, these symptoms must occur in the context of exposure to a potentially life-threatening event [5]. The traumatic event itself must involve an actual or threatened life-threatening situation, severe injury, or sexual violence [5]. To meet the criteria defined in the Diagnostic and Statistical Manual of Mental Disorders, Fifth Edition, Text Revision (DSM-5-TR), individuals must experience intrusive symptoms related to the event, engage in persistent avoidance of stimuli associated with the event, and display negative alterations in cognition, mood, arousal, and reactivity for a minimum of one month [5].

Aboulomania, a mental disorder characterized by pathological indecisiveness, is not included in the DSM-5-TR. It is defined by an individual's profound difficulty or inability to make choices in their daily life [6]. This condition significantly impairs social functioning, making it challenging to maintain personal and familial relationships [6,7]. This case report aims to explore the nature of aboulomania and its connection to other mental illnesses.

The term "aboulomania" was coined in 1883 by William Alexander Hammond, a military physician and neurologist. He described it as a form of insanity characterized by a lack of willpower, inertness, torpor, or paralysis of will [5-7]. In our everyday lives, we regularly make countless minor decisions. However, individuals with aboulomania experience severe indecisiveness that severely hampers their ability to function normally.

This case report focuses on an individual diagnosed with aboulomania, an extreme indecisiveness disorder. It sheds light on an alternative mental disorder that is not currently included in the DSM-5-TR. In addition, the report examines how aboulomania can manifest in conjunction with other mental illnesses listed in the DSM-5-TR. It also emphasizes the significant variation in symptomatology observed in PTSD and highlights the role of indecisiveness within this specific disorder.

## Case presentation

Subjective

The patient is a 40-year-old Caucasian male with a history of PTSD and cannabis use disorder. He has no known medical history, is single, and does not have any children. The patient presented for evaluation after his housing facility petitioned him, which led to a consultation with a psychiatrist. Notably, the patient had a previous suicide attempt two years ago when he cut his wrists, resulting in loss of consciousness due to blood loss. 

The patient's chief complaint was, "I do not know why I am here." Throughout the interview, the patient displayed guarded and evasive behavior. He remained unclear about recent substance use and expressed fear of potential repercussions. When asked to rate his mental health on a scale from 1 to 10, he found it challenging to quantify. However, he acknowledged feeling irritable. The writer mentioned staff reports from the patient's previous housing facility, including increased aggression, which upset and made him paranoid. He vehemently denied these claims. The patient expressed frustration with staff when they attempted to assign him a room near the nursing station. He pondered the treatment options available during his stay and expressed a desire to make the most of his time there. There was ongoing debate with the writer regarding the need for substance use treatment and his reluctance to consider it or explore other alternatives. Despite contrary collateral information, he adamantly denied substance use. The patient shared that he has spent the past two years alone at home, lacking interest in many activities and struggling to form close relationships. For the psychiatric review of systems, the patient experienced hypervigilance and exhibited startle responses, but he denied experiencing flashbacks or nightmares. He reported intrusive thoughts and frequently avoided leaving his house. There was no current indication of suicidal ideation, homicidal ideation, or self-injurious behavior, and he denied paranoia, preoccupations, and auditory or visual hallucinations. He stated that his appetite is normal and his sleep is "okay." He described his mood as "okay" but was unsure about his level of anxiety. He denied any symptoms of mania.

Objective

On mental examination, the patient appeared to be his stated age. He has short hair and a tall, thin body habitus. The patient spoke in a low volume with a normal rhythm, but his tone was monotonous. He exhibited spontaneous speech and was fluent in English. The patient's behavior was evasive, guarded, anxious, odd, and bizarre. The patient described his mood as "okay."The patient displayed a flat affect with a limited range. The patient's thought process was circumstantial but coherent. The patient engaged in debates about the type of treatment he wants and whether or not he believes he needs it. The patient's associations were intact. There were no indications of delusions, preoccupations, or paranoid thinking. The patient denied experiencing auditory or visual hallucinations, as well as any thoughts of self-harm, harm to others, or suicidal ideation. The patient appeared suspicious when questioned. The patient's judgment and insight were poor, as demonstrated by previous suicide attempts and substance use. He expressed significant ambivalence regarding treatment options and strongly denied any drug use. The patient was awake, alert, and oriented to person, place, and time. The patient's memory appeared intact for the purposes of the conversation. The patient's fund of knowledge was average. The patient's blood pressure was 130/77 mmHg, and his pulse was 62 beats/min; he was afebrile and had a respiratory rate of 16 breaths/ min. 

The patient's labs were non-contributory, except low vitamin D25 levels [27.7]. His urine drug screen (UDS) was negative, and his ethanol level was below 10. The patient denied using tobacco, and the Alcohol Use Disorders Identification Test (AUDIT-C) assessment yielded negative results. He also denied recent use of cannabis or any other illicit drugs, and the UDS conducted in the emergency department was negative. The patient had a chronic back pain condition that was post-back surgery. The patient denied having any family history of mental illness or suicide. The patient had previously undergone medication trials with sertraline (unknown dose or duration) for mood, clonazepam (unknown dose or duration)​​​​​​ for anxiety​, and quetiapine (unknown dose or duration) for sleep and mood. Currently, he is not taking any psychiatric medications and does not have regular outpatient follow-up. He expressed hesitancy about starting any medications during his inpatient stay, displaying paranoia about our intentions and stating that he does not believe he requires medication. The patient is single and does not have any children. He is currently unemployed and has no legal issues. He denied experiencing any psychological or childhood trauma. In 2006, he enlisted in the army and was involved in combats. He witnessed the death of his friend in front of him. He denied any family history of psychiatric illnesses. 

Assessment

The patient is a 40-year-old Caucasian male with a history of PTSD and cannabis use disorder. He has no known medical history, is single, and does not have any children. The patient presented for an evaluation after being petitioned. He was exhibiting symptoms of worsening anxiety associated with PTSD. He was psychiatrically decompensated and met the 401 criteria for inpatient psychiatric hospitalization. 

Plan

The patient was involuntarily committed due to suicidal ideations, worsening mood lability, and impulsivity. He was started on mirtazapine 7.5 mg nightly for mood improvement.

## Discussion

Risk factors and symptoms of aboulomania

Aboulomania is associated with specific parental styles, particularly among individuals who are biologically predisposed to the disorder [8,9]. Overprotective or authoritarian parenting styles, as well as excessive involvement or intrusive behaviors from the primary caregiver, can foster dependence in the child [9,10]. These types of parental behaviors create uncertainty and doubt in children, which can impede their ability to function independently as adults and lead them to feel incapable of living autonomously [11]. Furthermore, many individuals with aboulomania have experienced social humiliation or bullying during their developmental years [12]. Anxiety and depression are also closely associated with aboulomania [6].

Aboulomania is associated with several symptoms that can indicate its presence [6-12]. These symptoms include experiencing high levels of uncertainty and anxiety, feeling anticipatory anxiety when faced with decision-making tasks that often result in mental blocks, avoiding personal responsibility by evading decision-making altogether, engaging in lengthy decision-making processes, struggling to function independently or make decisions without relying heavily on the support of others, and engaging in excessive analysis of situations. These symptoms collectively illustrate the challenges and patterns commonly observed in individuals affected by aboulomania.

Examples of symptoms stated by this patient are as follows: "At times, it is challenging to make decisions because I feel like I have limited information. It's similar to going to a National Park and trying to choose a hiking trail, but you only have a small amount of information about each trail." "I constantly feel the need for more information before making decisions."

Normal indecisiveness versus pathological indecisiveness

To what extent will people go to avoid making decisions? And when does it become pathological? In his book "The Paradox of Choice" published in 2005, Barry Schwartz explored the notion that having more options does not necessarily lead to better decision-making; in fact, it can often make decisions more challenging. According to Schwartz et al., an abundance of choices can create a psychological barrier that demotivates individuals [13]. This is supported by the choice overload hypothesis, which suggests that while having choices initially seems desirable, beyond a certain threshold, it becomes overwhelming and leads to decreased motivation or a desire to do everything [13]. When accompanied by excessive anxiety, depression, or neuroticism, this phenomenon becomes pathological [14].

Excessive anxiety can contribute to indecisiveness, and the opposite is also likely. Research indicates that individuals with high levels of neuroticism tend to score higher on tests assessing indecisiveness [1-6]. It is normal to experience a certain level of indecisiveness in our everyday lives. However, when symptoms become clinically significant and cause distress and impairment in a person's functioning, they can be considered a part of a mental disorder [3]. The development of aboulomania likely involves a combination of environmental factors experienced during development and biological factors [6]. Prominent symptoms associated with anxiety and mood disorders are often observed in aboulomania. The causes of aboulomania are likely multifactorial and can involve brain circuitry, personality, psychosocial stressors, trauma, and/or poor coping skills.

Associations with neuroanatomy**

The decision-making process involves communication between the prefrontal cortex and hippocampus [14,15]. The prefrontal cortex has the capacity to hold multiple pieces of information simultaneously [14,15]. However, this ability can sometimes overwhelm individuals when faced with decisions, regardless of their significance. They may excessively analyze each situation, experiencing paralysis by analysis and generating concerns about potential negative outcomes. This obsession with information scarcity, difficulty in assessing value, and uncertainty of outcomes can be prominent in individuals with aboulomania [8].

The prefrontal cortex is directly implicated in aboulomania [14,15]. Research has shown that individuals with damage to their prefrontal cortex exhibit poor decision-making abilities [14,15]. It is speculated that individuals with aboulomania have irregular functioning in their prefrontal cortex, leading to an obsession with overanalysis and uncertainties about decision outcomes [14,15].

Diagnosis and treatment of abulomania 

Motivational interviewing, reflective listening, goal setting, and exploration are crucial therapeutic approaches for aboulomania. The main objective is to evoke and strengthen the individual's motivations for change, while therapists respond with empathic understanding rather than confronting the lack of change. Psychotherapy serves as the primary treatment method for aboulomania, aiming to enhance individuals' activity and independence. Through therapy, troubling emotions, thoughts, and behaviors are identified and specifically addressed. The treatment approach involves a combination of medications and therapy to target underlying symptoms.

In order to diagnose aboulomania, mental health professionals need to first eliminate any organic factors that may be causing the symptoms because there are no specific laboratory tests available for this purpose [6]. However, psychiatrists have a range of assessment tools that they can utilize to evaluate aboulomania [12]. These tools include the Minnesota Multiphasic Personality Inventory (MMPI), Millon Clinical Multiaxial Inventory - Fourth Edition (MCMI-IV), Rorschach Psychodiagnostic Test, and Thematic Apperception Test (TAT). 

Psychotherapy is the preferred treatment method for aboulomania to address symptoms that may resemble those of obsessive-compulsive disorder (OCD), anxiety, or depression [6,13]. It is important to take small, tangible steps and help patients make choices without overwhelming them, a concept known as choice closure and acceptance.

In the present case report, the patient initially hesitated to express his desire to engage and struggled to identify actionable steps toward his treatment goals. He used uncertainty about his admission as a way to avoid exploring and refining his statements. Through the use of reflective listening and concrete goal setting, the patient was able to make tangible choices without feeling overwhelmed.

PTSD

In the United States, the estimated lifetime risk of developing PTSD by the age of 75 is 8.7% [3]. The diagnosis of PTSD requires meeting specific criteria, which include (a) experiencing or being threatened with death, serious injury, or sexual violence; (b) the presence of intrusive symptoms related to the traumatic event; (c) persistent avoidance of stimuli associated with the trauma; (d) negative changes in thoughts and mood; and (e) significant alterations in arousal and reactivity [3]. The above is not all encompassing and does not include specifiers or the breakdown of symptoms within each criteron. 

The likelihood of developing PTSD following a similar level of trauma varies among different cultural groups, such as those with fatalistic or self-blaming beliefs [3]. Symptoms typically appear within the first three months after the traumatic event, but they can also be delayed for years before meeting the diagnostic criteria, known as delayed expression [3].

Risk Factors for PTSD 

The risk factors for PTSD can be divided into three categories: pre-traumatic, peri-traumatic, and post-traumatic factors. Pre-traumatic factors include childhood emotional problems and prior trauma experiences [1]. More pre-traumatic risk factors include lower socioeconomic status and previous exposure to trauma [1]. Furthermore, biologic and genetic factors play a role, such as being a female and having a strong family history [3].

During the traumatic event itself, known as peri-traumatic factors, various elements can impact the subsequent psychological response. Environmental factors, such as the severity of the trauma, perceived life threat, personal injury, military service, witnessing atrocities, or being involved in combat situations, significantly influence the psychological aftermath [2]. 

Post-traumatic factors can be subdivided into temperamental and environmental risk factors. Temperamental factors include poor coping mechanisms and negative appraisals of the traumatic experience [3]. Negative appraisals involve avoiding reminders of the traumatic experience [3]. In addition, environmental factors, such as repeated exposure to distressing reminders or frequent adverse life events after the trauma, contribute to the persistence of trauma-related symptoms [3]. 

Understanding these pre-traumatic, peri-traumatic, and post-traumatic factors is crucial for mental health professionals when assessing and treating individuals who have experienced trauma. By recognizing and addressing these factors, tailored interventions and support can be provided [3].

Treatment of PTSD

Cognitive-behavioral therapy (CBT) is widely recognized as the most effective treatment for PTSD. In CBT, the focus is on addressing the traumatic events themselves and recognizing how cognitive distortions lead to the patient's behavior. The goal of this therapy is to modify these negative associations and change perceptions [3].

There are four FDA-approved medications for PTSD, which include selective serotonin reuptake inhibitors (SSRIs) and serotonin-norepinephrine reuptake inhibitors (SNRIs). These medications include sertraline, paxil, fluoxetine, and Effexor.

Other mental illnesses associated with aboulomania 

Aboulomania has been found to be associated with other mental illnesses, such as depression and OCD [6-12]. There is a significant overlap between aboulomania and OCD. Despite the overlap of symptoms among other mental illnesses, aboulomania is not recognized as a specific disorder in the DSM-5-TR. This highlights the limitations of the DSM-5-TR as a comprehensive diagnostic tool and emphasizes the need for additional information beyond its guidelines. Specifically, individuals with PTSD often experience symptoms of depression or anxiety, which are closely linked to aboulomania.

Review of the current literature 

Further exploration is necessary due to the limited research conducted in this area. There was some research completed by Frost and Shows, which explains that indecisiveness appears to be a symptom of OCD. Pathological indecisiveness appears to be correlated with dimensions of perfectionism and with compulsive hoarding [16]. In addition, indecisiveness appears to be associated in a variety of life domains (social, academic, family, and daily life) [16]. Moreover, OCD appears to coincide with pathological indecisiveness and intolerance of uncertainty [17]. Individuals with OCD show elevated intolerance of uncertainty, but only when outcome probabilities are themselves uncertain [17]. Future research focused on how aboulomania is associated with mental illnesses as a symptom needs to be further explored. Another study examined how PTSD itself can cause indecisiveness, especially in acute situations. It appears that individuals experiencing PTSD symptoms reported high levels of acute stress when faced with high acuity situations [18]. Acute stress in these studies was associated with performance deficits on complex cognitive tasks, verbal memory impairment, and heightened assessment of risk [18].

## Conclusions

This case report focuses on a male patient with aboulomania, an uncommon mental illness that is not included in the DSM-5-TR. The authors aim to shed light on the association between aboulomania and other medical conditions. In addition, this report highlights the distinction between aboulomania and PTSD, emphasizing the need for clinicians to consider both conditions in their treatment and diagnostic decisions. Furthermore, the case report raises awareness about the limitations of the DSM-5-TR and emphasizes the importance of maintaining an open-minded approach when treating mental illnesses.
